# A novel rapid measurement of hallux valgus parameters using the built-in photo edit function of smartphones

**DOI:** 10.1186/s12891-021-04604-y

**Published:** 2021-08-21

**Authors:** Tianji Huang, Lin Wang, Chao Lu, Weiyang Zhong, Zenghui Zhao, Xiaoji Luo

**Affiliations:** Department of Orthopedic Surgery, The First Affiliated Hospital of Chongqing Medical University, Chongqing, 400016 People’s Republic of China

**Keywords:** Smartphone, Rapid measurement, Hallux valgus, HVA, IMA

## Abstract

**Objective:**

The objective of this study was to assess the accuracy and reliability of and time taken by a novel method using the built-in photo-edit function of smartphones compared with PACS in measuring hallux valgus parameters.

**Methods:**

Seventy patients (124 ft) admitted to our hospital with a diagnosis of hallux valgus without previous surgical procedures were retrospectively reviewed. The foot radiographs of all the patients were extracted from PACS. The hallux valgus angle (HVA) and the first and second intermetatarsal angles (IMAs) were measured by PACS and by this novel method using the built-in photo-edit function of a smartphone. The results of these two methods were compared, and the accuracy and reliability were assessed between these two methods.

**Results:**

The average parameters measured by PACS were as follows: HVA average: 37.43 ± 9.61°; IMA average: 13.37 ± 4.01°. The average parameters measured by smartphones were as follows: HVA average: 37.09 ± 9.52° and IMA average: 13.49 ± 3.91°. When compared by the independent-samples T test, the average parameters between PACS and smartphones were not significantly different (HVA PACS vs HVA smartphones: *P* = 0.776; IMA PACS vs IMA smartphones: *P* = 0.816). The variability of the HVA (F = 0.166, *P* = 0.992) and IMA (F = 0.215, *P* = 0.982) measurements was similar for the PACS and smartphones. The ICCs of the average parameters of four measurements of HVA and IMA between PACS and smartphones were 0.995 (0.991–0.997) and 0.970 (0.958–0.979), indicating that the two methods were highly correlated. For the smartphone measurement, the interobserver and intraobserver reliability was very good for HVA and IMA. The average measurement time of PACS was 25.41 ± 0.86 s, and the average measurement time of smartphones was 20.29 ± 1.22 s. The smartphone time was significantly faster than that of PACS by approximately 5 s (*P*<0.001).

**Conclusion:**

This novel method using the built-in photo-edit function of smartphones is accurate, reliable, convenient and time-saving in measuring the angles of hallux valgus.

## Introduction

Measuring the angles on radiographs is a valuable tool to diagnose and evaluate hallux valgus. These angles may be used to classify the severity of hallux valgus and guide appropriate management decisions [1]. Some of the valuable angles in assessing hallux valgus include the hallux valgus angle (HVA), the first and second intermetatarsal angle (IMA), the distal metatarsal articular angle (DMAA), the distal articular set angle (DASA), the hallux interphalangeus angle (IPA), the distal medial cuneiform angle (DMCA) and the metatarsus adductus angle (MAA) [2]. Traditionally, these angles are measured by using a marker pen and a protractor on radiograph film. This manual method takes much time and is error prone [3, 4]. Picture archiving and communication systems (PACS) have been increasingly commonly used as the gold standard for angle measurements in orthopaedic radiographs. However, PACS of certain hospitals may not be used in other hospitals, and it is not convenient for doctors to communicate online with each other about the radiographs. Some iPhone apps have been introduced to measure the angles of hallux valgus [5–8]. Even though such apps are reliable and time-saving, there are still some shortcomings, such as lack of maintenance and updates, lack of fit for the newest smartphones, bugs or extra payments, and lack of fit for the Android system.

In this study, we used a novel rapid method with the built-in photo-edit function of Android smartphones to measure hallux valgus parameters. We assessed the accuracy and reliability of and time taken by this novel method and PACS in measuring hallux valgus parameters.

## Materials and methods

All methods were carried out in accordance with relevant guidelines and regulations. This study was approved by the ethics committee of The First Affiliated Hospital of Chongqing Medical University. Informed consent was obtained from all patients.

### Patients selection

Seventy patients (124 ft) admitted to our hospital with a diagnosis of hallux valgus without previous surgical procedures were retrospectively reviewed. Anteroposterior weight-bearing radiographs were taken with the X-ray beam centred on the midfoot and inclined at 20° from vertical in the sagittal plane at a distance of 100 cm. The foot radiographs of all the patients were extracted from PACS.

### Measure methods

**Fig. 1:**
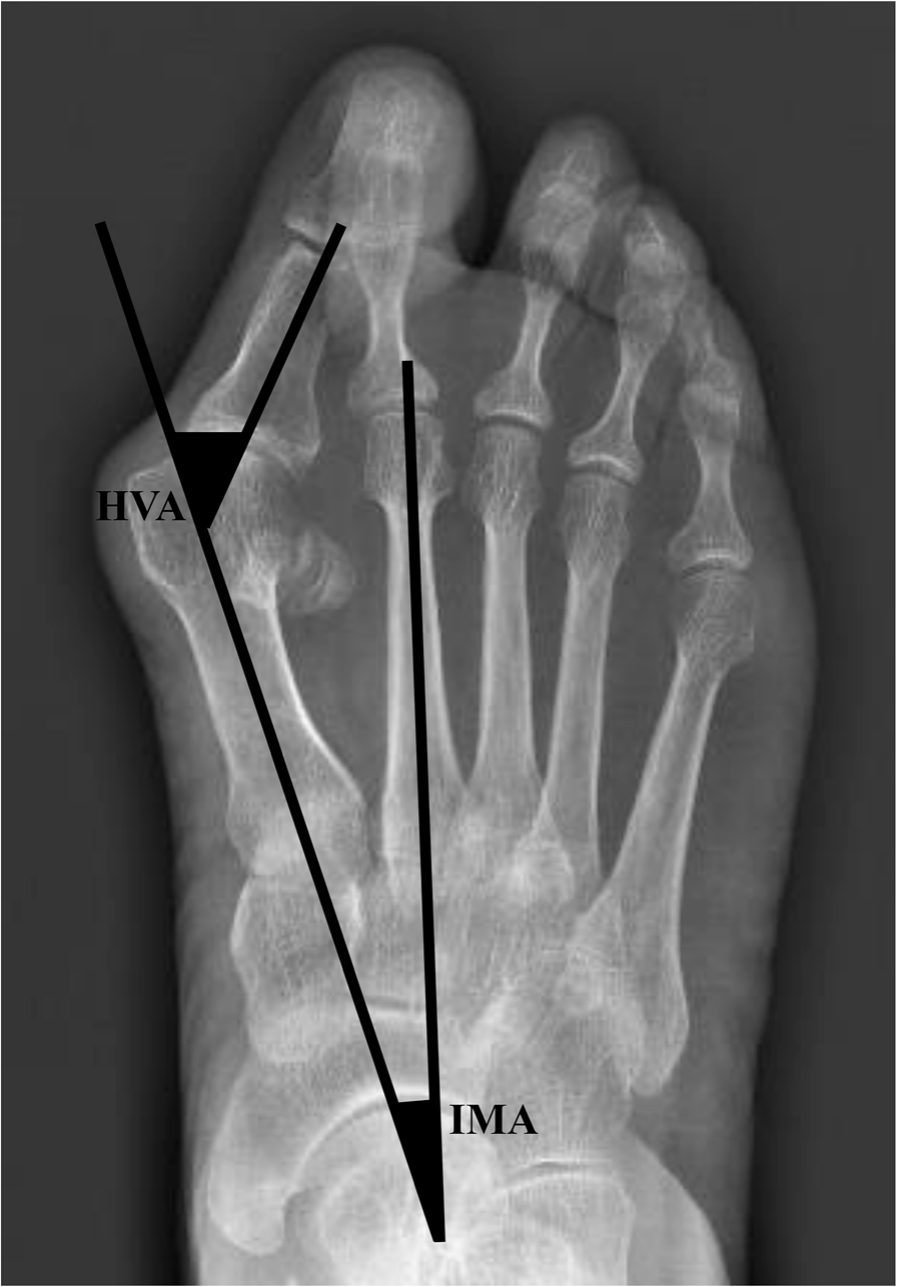
HVA and IMA were measured according to the description of the ad hoc committee of the American Orthopaedic Foot and Ankle Society

The hallux valgus angle (HVA) and the first and second intermetatarsal angles (IMAs) were measured according to the description of the ad hoc committee of the American Orthopaedic Foot and Ankle Society [9] (Fig. 1).

Measurements by PACS were performed with the intrinsic tool in PACS to mark and measure the HVA and IMA.

**Fig. 2:**
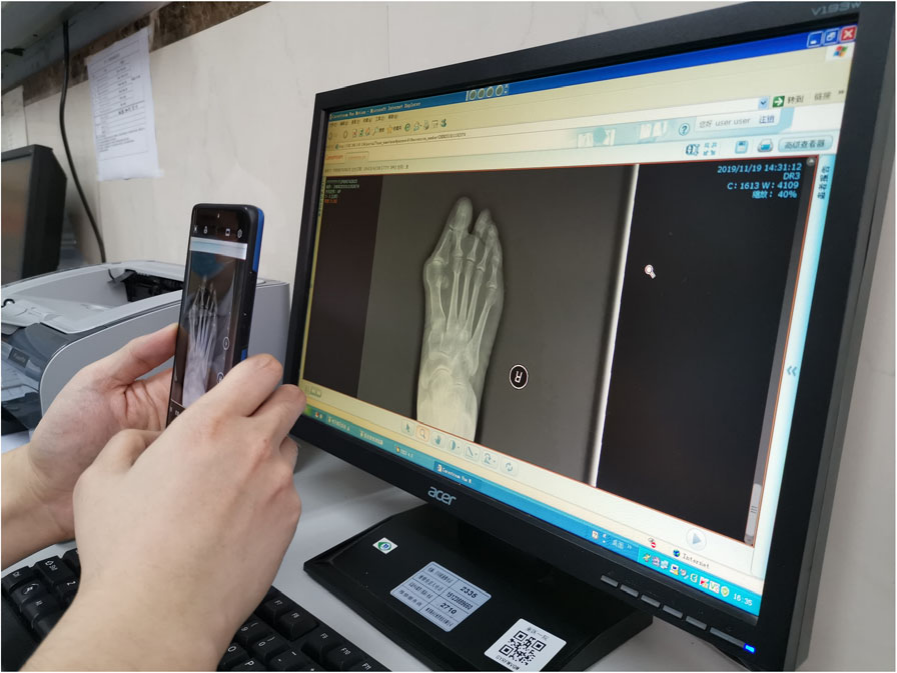
The plane of the smartphone screen should be parallel to the computer screen during the photo acquisition to eliminate parallax errors

**Fig. 3:**
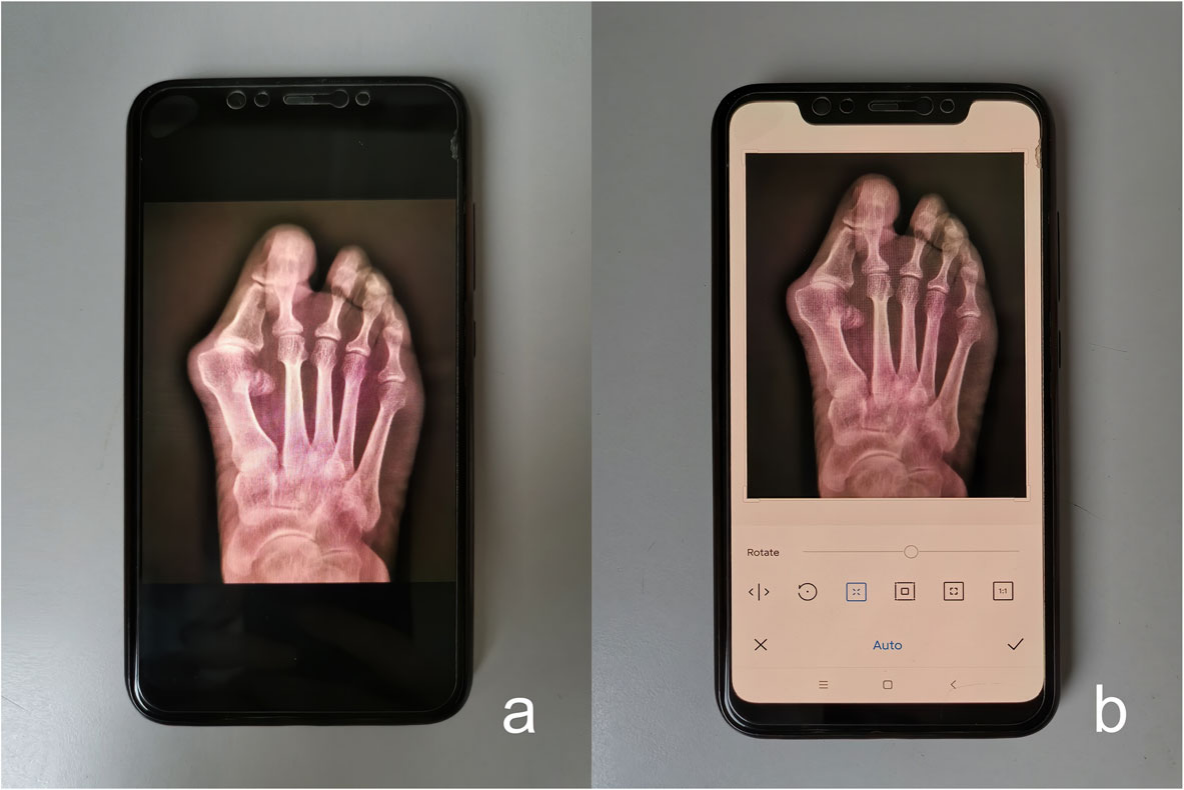
**a**. The targeted photo was shown on the smartphone. **b**. The intrinsic photo-editing system was used to rotate the photos of radiographs

**Fig. 4:**
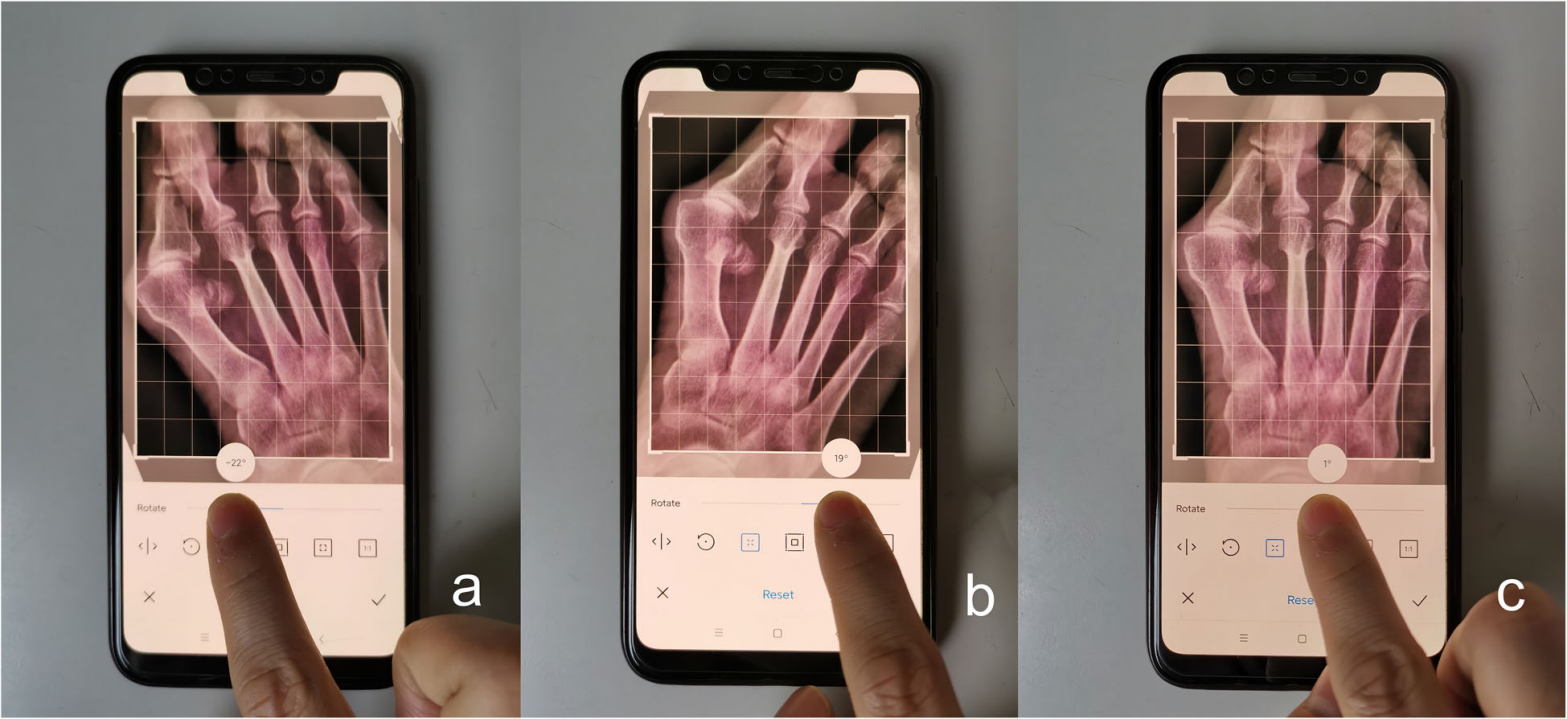
Measurement of HVA and IMA. **a**. Rotate the photo until the long axis of the first proximal phalanx is parallel to or overlaps the vertical grid lines, and record the angle at this time. **b**. Rotate the photo until the long axis of the first metatarsal bone is parallel to or overlaps the vertical grid lines, and record the angle at this time. **c**. Rotate the photo until the long axis of the second metatarsal bone is parallel to or overlaps the vertical grid lines, and record the angle at this time

Observers used the built-in camera of Android smartphones to take pictures without any marks. The plane of the smartphone screen was kept parallel to the computer screen during the photo acquisition to eliminate parallax errors (Fig. 2). The intrinsic photo-editing system was used to rotate the photos of the radiographs, allowing the rotation angle scale and grid lines to be seen simultaneously and clearly (Fig. 3). For measurement of the HVA, first, the photo was rotated until the long axis of the first proximal phalanx was parallel to or overlapping the vertical grid lines, and then, the angle was recorded; second, the photo was rotated until the long axis of the first metatarsal bone was parallel to or overlapping the vertical grid lines, and then, the angle was recorded; finally, the value of the difference between these two angles that represented the HVA was recorded (Fig. 4). The IMA measurement was similar, first, the photo was rotated until the long axis of the first metatarsal bone was parallel to or overlapping the vertical grid lines, and then, the angle was recorded; second, the photo was rotated until the long axis of the second metatarsal bone was parallel to or overlapping the vertical grid lines, and then, the angle was recorded; finally, the value of the difference between these two angles that represented the IMA was recorded (Fig. 4).

Two attending physicians (two of the authors) in the orthopaedic department measured the radiographs independently. To minimize the deviation, each angle obtained by PACS and smartphones was measured twice over two weeks. The measurement order of the two observers was as follows:

- Week 1: The two observers measured the HVA and IMA using PACS (first time).
- Week 3: The two observers measured the HVA and IMA using smartphones (first time).
- Week 5: The two observers measured the HVA and IMA using PACS (second time).
- Week 7: The two observers measured the HVA and IMA using smartphones (second time).

The order of the patients was randomized to reduce possible recall by using the “rand-function” of the Excel software. The time of each measurement was recorded with a stopwatch. A total of 992 (124 ft × 2 observers × 2 times × 2 methods) HVAs and 992 IMAs were recorded in Excel 2016. The average value of each angle was calculated.

### Statistic analysis

Statistic analysis was performed blinded. The statistical software SPSS 21.0 (SPSS, Inc., Chicago, IL, USA) was used to analyse the data. Data are presented as “mean ± standard deviation”. An independent-samples T test (if the values were normally distributed, checked by the Kolmogorov-Smirnov test) was used to compare the difference between the average parameters of the two measurement methods and to analyse the difference between times of the two measurement methods. One-way analysis of variance was used to compare the differences between different times of measurement. Two-way random intraclass correlation coefficients (ICCs) were used to analyse the reliability of the two measurement methods. Poor reliability was considered with values of 0 to 0.20, fair reliability with values of 0.21 to 0.40, moderate reliability with values of 0.41 to 0.60, substantial or good reliability with values of 0.61 to 0.80, and nearly perfect or very good reliability with values of 0.81 to 1.0 [8].

## Results

The present study group comprised 70 patients (61 females and 9 males). The age of these patients was 60.76 ± 13.39 years (ranging from 23 to 83 years). A total of 124 ft consisting of 59 left feet and 65 right feet were reviewed. The average parameters measured by PACS were as follows: HVA average: 37.43 ± 9.61°; IMA average: 13.37 ± 4.01°. The average parameters measured by smartphones were as follows: HVA average: 37.09 ± 9.52° and IMA average: 13.49 ± 3.91°. When compared by independent-samples T tests, the average parameters between PACS and smartphones were not significantly different (HVA PACS vs HVA smartphones: *P* = 0.776; IMA PACS vs IMA smartphones: *P* = 0.816). Therefore, smartphones could also be used as well as PACS to measure the two angles when diagnosing hallux valgus. The variability of HVA (F = 0.166, *P* = 0.992) and IMA (F = 0.215, *P* = 0.982) measurements was similar for the PACS and smartphones (Table 1). The ICCs of the average parameters of four measurements of HVA and IMA between PACS and smartphones were 0.995 (0.991–0.997) and 0.970 (0.958–0.979), indicating that the two methods were highly correlated. The concordance between PACS and smartphone measurements was very good for the HVA and IMA of every measurement (Table 2). For the smartphone measurement, the interobserver and intraobserver reliability was very good for HVA and IMA (Table 3). The average measurement time of PACS was 25.41 ± 0.86 s, and the average measurement time of smartphones was 20.29 ± 1.22 s. The smartphone time was significantly faster than that of PACS by approximately 5 s (*P*<0.001).

**Table 1:** Mean ± SD values of all measurements and their comparison

	HVA (°)	IMA (°)
PACS A t1	37.08 ± 9.53	13.32 ± 4.18
PACS A t2	37.12 ± 9.75	13.12 ± 4.13
PACS B t1	37.83 ± 9.73	13.66 ± 4.01
PACS B t2	37.69 ± 9.71	13.38 ± 4.25
Smartphone A t1	36.87 ± 9.44	13.35 ± 4.29
Smartphone A t2	37.45 ± 9.57	13.55 ± 4.08
Smartphone B t1	36.98 ± 9.77	13.58 ± 3.90
Smartphone B t2	37.04 ± 9.61	13.48 ± 4.00
Significance (*P* value)	0.992	0.982

*P* values indicate the significance between the values in the same column

PACS A t1: the results of the first time of observer A using PACS, and so on

Smartphone B t2: the results of the second time of observer B using smartphones and so on

**Table 2:** Reliability of the results using PACS and smartphones for every measurement

	ICC (95% CI)	*P* value
Observer A t1 PACS versus smartphones		
HVA	0.983 (0.975–0.988)	0.000
IMA	0.889 (0.846–0.921)	0.000
Observer A t2 PACS versus smartphones		
HVA	0.981 (0.972–0.986)	0.000
IMA	0.895 (0.851–0.926)	0.000
Observer B t1 PACS versus smartphones		
HVA	0.983 (0.963–0.991)	0.000
IMA	0.929 (0.901–0.950)	0.000
Observer B t2 PACS versus smartphones		
HVA	0.984 (0.973–0.990)	0.000
IMA	0.926 (0.896–0.947)	0.000

**Table 3:** Inter- and intraobserver reliability of observer A’s versus observer B’s measurements using smartphones

	ICC (95% CI)	*P* value
Observer A t1 versus observer B t1		
HVA	0.974 (0.962–0.981)	0.000
IMA	0.867 (0.816–0.905)	0.000
Observer A t2 versus observer B t2		
HVA	0.981 (0.972–0.987)	0.000
IMA	0.894 (0.852–0.924)	0.000
Observer A t1 versus observer A t2		
HVA	0.971 (0.958–0.980)	0.000
IMA	0.904 (0.866–0.932)	0.000
Observer B t1 versus observer B t2		
HVA	0.991 (0.987–0.994)	0.000
IMA	0.943 (0.920–0.960)	0.000

Observer A t1: the results of the first time of observer A, and so on

Observer B t2: the results of the second time of observer B, and so on

## Discussion

Measurements of angles are important in diagnosing and evaluating hallux valgus. The surgical indication and choice of surgical methods depend on the clinical symptoms and radiographic measurement of certain angles. Some of the valuable angles in assessing hallux valgus include HVA, IMA, DMAA, DASA, IPA, DMCA and MAA. Because of the similar steps in measurement, HVA and IMA were chosen to be measured on behalf of other angles in this study.

These angles are traditionally measured by using a marker pen and a protractor on radiograph film, which is time consuming and error prone [3, 4]. Computer-assisted measurements of digital images have been widely used and have been shown to be more accurate and efficient [10, 11]. PACS have been increasingly commonly used, have the advantages of economical storage and easy access, and can measure the angles of digital radiographs by intrinsic software. However, not every hospital in developing countries has PACS, and different hospitals may have different kinds of PACS that cannot be seen and operated on computers at other hospitals. It often happens that patients come to the outpatient department with radiograph films from other hospitals that cannot be seen and manipulated on the computers at the present hospital; consequently, doctors have to measure the angles by traditional methods with marker pens and protractors, which may not always be carried around by physicians.

Smartphones are very popular, and nearly every doctor has one. These devices are small and easy to carry. Smartphones are very convenient for measuring all angles of bones by promptly shooting photos of radiograph films. In addition, smartphones have powerful social communication functions. Orthopaedic surgeons can discuss and communicate with each other about patients’ diagnosis and treatment via emails or apps such as WeChat (a popular Chinese social media messaging app) using smartphones. Remote consultations can also be implemented by smartphone apps.

Measurement of the angles of orthopaedic radiograph films by smartphones has been used in many kinds of orthopaedic diseases [12–14]. For the measurement of angles in hallux valgus patients, some smartphone apps have been introduced. The iPhone Hallux Angles app was proven to be a reliable method to measure HVA and IMA when compared to PACS [6]. The Tiltmeter software for iPhones is precise and less time-consuming than traditional measurements with protractors [7]. One app named iPinPoint is suggested to be reliable for the measurement of HVA and IMA, even for nonexperienced users [8]. Even though these apps are reliable and time-saving, there are some shortcomings. Due to a lack of maintenance and updates, some of these apps are not compatible with the newest smartphones. Moreover, these apps are not as stable and reliable as the built-in photo-edit function of the system and may have bugs or requirements for extra payment. Most of these apps are built for the iPhone but are not fit for the Android system.

In this study, we presented a novel rapid measurement of hallux valgus parameters using the built-in photo-edit function of smartphones. The difference in the results of angle parameters measured by smartphones and PACS were studied via different kinds of statistical methods. The time differences of the two measurements were also compared. As with a previous similar measurement method of smartphones used in spine-pelvic sagittal balance parameters [15], in this study, the results of smartphones were highly consistent with those measured by PACS. Furthermore, the interobserver and intraobserver reliability was very good for the smartphone method, indicating that this novel measurement using the built-in photo-edit function of smartphones is precise and reliable in measuring HVA and IMA in hallux valgus patients. Furthermore, the results showed that the smartphone time was significantly faster than that of PACS by approximately 5 s.

According to this study, we concluded that this novel smartphone method is a more convenient and faster method to measure hallux valgus parameter angles than PACS. In addition, the built-in photo-edit function of smartphones does not need to download special apps and is more stable without extra fees. Due to limited space, other parameters of hallux valgus were not discussed in this study. However, based on the similarity of the measurement steps, this smartphone method may also be used to measure other parameters of hallux valgus. However, this approach also has some potential errors, such as the difference in foot position when taking the X-ray and different reference lines of a certain angle among different observers. Nonetheless, these potential errors are common not only in smartphone measurements but also in PACS or manual measurement methods. It is worth noting that if the camera of the smartphone is not parallel to the radiograph, there may be intrinsic error due to the deformed picture. However, this error could be minimized by the user holding the smartphone paralleled to the radiographs when measuring [8].

## Conclusion

This novel method using the built-in photo-edit function of smartphones is accurate, reliable, convenient and time-saving in measuring the angles of hallux valgus.

## Data Availability

The datasets used or analysed during the current study are available from the corresponding author on reasonable request.
